# Control blindness: Why people can make incorrect inferences about the intentions of others

**DOI:** 10.3758/s13414-016-1268-3

**Published:** 2017-01-11

**Authors:** Ashlan S. Willett, Richard S. Marken, Maximilian G. Parker, Warren Mansell

**Affiliations:** 1 Department of Cognitive Science, Vassar College, Poughkeepsie, New York 12604 USA; 2 Department of Psychology, Antioch University, Los Angeles, USA; 3 School of Health Sciences, University of Manchester, 2nd Floor Zochonis Building, Brunswick Street, Manchester, M13 9PL UK

**Keywords:** Control theory, Theory of mind, Intentional state, Inference

## Abstract

There is limited evidence regarding the accuracy of inferences about intention. The research described in this article shows how perceptual control theory (PCT) can provide a “ground truth” for these judgments. In a series of 3 studies, participants were asked to identify a person’s intention in a tracking task where the person’s true intention was to control the position of a knot connecting a pair of rubber bands. Most participants failed to correctly infer the person’s intention, instead inferring complex but nonexistent goals (such as “tracing out two kangaroos boxing”) based on the actions taken to keep the knot under control. Therefore, most of our participants experienced what we call “control blindness.” The effect persisted with many participants even when their awareness was successfully directed at the knot whose position was under control. Beyond exploring the control blindness phenomenon in the context of our studies, we discuss its implications for psychological research and public policy.

An important domain of psychological research is the study of inferences regarding the goal states of individual agents, which may be referred to as their intentions, goals, or purposes. A classic study showed that people attribute intention to the unintentional behavior of animated geometric shapes (Heider & Simmel, 1994). It is well established that humans and some other animals have a tendency to take “the intentional stance” (Dennett, 1989) and to regard the actions they observe in others as intentional, connecting them to specific goals (Gergely, Nádasdy, Csibra, & Bíró, 1995; Kiley Hamlin, Ullman, Tenenbaum, Goodman, & Baker, 2013). This propensity may result in a bias to judge a wide range of unintentional behaviors as intentional (Rosset, 2008; Rosset & Rottman, 2014). This bias calls into question the ability to *accurately* detect the true goal of another’s behavior. Some studies purport to show good levels of accuracy (Barrett, Todd, Miller, & Blythe, 2005; Call & Tomasello, 2008). However, these studies did not measure a person’s ability to distinguish behavior that is *actually* intentional from behavior that is not (Pantelis & Feldman, 2012). Rather, the behavior to be judged was either generated by people acting *as though* they were behaving with a certain intent or by programming apparently intentional behavior using computer animation. To correctly measure the accuracy of inferences about a person’s intent, an objective basis is needed for distinguishing intentional from unintentional behavior (Marken, 2013a). We will show that perceptual control theory (PCT) provides the theoretical basis for making this distinction (Powers, 1973).

The origins of PCT trace back to the 1950s and 1960s, within the field of control engineering (Powers, Clark, & McFarland, 1960), and it acknowledges influences from the early cybernetic movement (Ashby, 1958; Wiener, 1948). Despite its age, the theory is somewhat consistent with the field of active inference (Kilner, Friston, & Frith, 2007) and contemporary embodied accounts of sensorimotor function (Carey, Mansell, & Tai, 2014) that in turn draw from early work in psychology on ideomotor theory (James, 1890). PCT has been supported by recent findings based on advances in methodology within the realms of human performance (Schaffer et al., 2013, 2015), comparative animal behavior (Bell, 2014; Bell, Bell, Schank, & Pellis, 2015), and basal ganglia function (Barter et al., 2015; Yin, 2014). We selected PCT over alternative modeling approaches for this study because it provides a parsimonious model of intentional behavior that has been applied across the life and social sciences (Mansell & Carey, 2015). Furthermore, it takes the philosophical stance, essential for the current study, that a person’s behavior has a “correct” goal that can be precisely modeled, despite being internal to the individual.

PCT identifies intentional behavior as *control*. Control is acting to bring variable aspects of the environment to pre-selected *reference states* and keep them there, protected from the effects of environmental disturbances. A person controlling the temperature of a shower, for example, can be seen to be acting with the intent of keeping the water at a comfortable temperature. This reference state is protected from disturbances, such as changes in the hot water pressure by other users.

According to PCT, intentional behavior is the control of perception within a closed loop, and this process entails a number of simultaneous functions (Powers, 1973; see Fig. 1). The control process involves comparison of a perceptual signal representing a variable aspect of the environment that is under control—such as shower water temperature—with a *reference signal* in the nervous system that specifies the intended state of this variable. Any difference between the perceptual signal and the reference signal produces an error signal, which drives system outputs—such as the turning of the hot water handle. These outputs “loop back” and have feedback effects via the environment that “push” the perceptual signal closer to the reference signal, thereby reducing the error signal. The result is that the perception—of shower water temperature, in this case—is maintained in a reference state, protected from disturbances by appropriate variations in system outputs. The perceptual aspect of the environment represented by the perceptual signal is, thus, kept under control; this is the nature of a *controlled variable*.

**Fig. 1:**
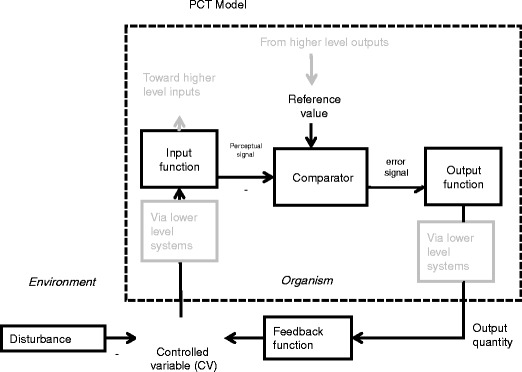
The PCT control unit shown with key terms and functional operations. The boxes denote functional operations that are applied to quantities within the environment or to signals within the organism. The *dotted box* denotes the organism–environment boundary. The *minus sign* denotes where a quantity is subtracted from the quantity passing around the loop. This single control loop is a functional simplification of a hierarchy of control loops that are represented in *gray*

An illustrative control task is known as the *rubber band demonstration* (Powers, 1973). It involves a demonstrator (D) and a volunteer (V), a whiteboard with a small dot marked in the middle, a figure-eight-shaped rubber band with a knot in the middle, and two pens (see Fig. 2). Both D (on the right) and V (on the left) hold pens within the opposite loops of the rubber band so that their movements are recorded on the whiteboard. First, D gives V an instruction that is not heard by the viewer. D and V then proceed to draw on the whiteboard, and the viewer is required to guess the instruction that V is following.

**Fig. 2:**
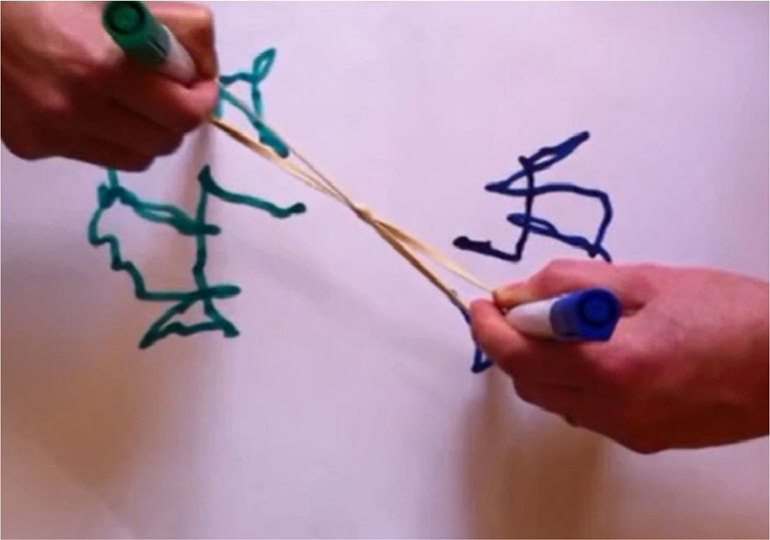
This is an image of a typical record of movements toward the end of the video demonstration

V has been instructed to keep the knot of the rubber band over a dot in the center of the page. The position of the knot is the variable to be controlled in this task and the dot specifies the reference state of this variable. From a PCT perspective, V has been instructed to adopt the intention of keeping the controlled variable (knot position) in a particular reference state (over the dot). V is engaging in control of the position of the knot relative to the dot. Therefore, asking the viewer of this demonstration to guess what V has been instructed to do is equivalent to asking for an inference regarding V’s intention.

According to PCT, V keeps the knot over the dot in order to match a reference signal that specifies that the perception of the distance from knot to dot should be maintained close to zero. Disturbances to the position of the knot, such as those produced by D’s pen movements, will result in error signals that drive compensating movements of V’s pen. These movements bring the knot back to the reference state. Because of the physics of the situation, V’s intention to keep the knot in the reference state (over the dot) is achieved only when V’s “compensating” pen movements are approximately equal and opposite to D’s “disturbing” pen movements (see Fig. 1).

The observed reference state of a controlled variable can be considered an intended result of an agent’s actions. Thus, in the rubber band demonstration, keeping the knot close to the dot is the intended result of V’s pen movements; it is what V intends to do. There are, however, many other results of V’s actions that are not intended. For example, the picture traced out by V’s pen movements, which looks a bit like a kangaroo boxing with another kangaroo (see left side of Fig. 1), is an unintended result of the actions that keep the knot over the dot. V did not intend to draw a boxing kangaroo. Nevertheless, in pilot studies, many viewers of V’s behavior claimed V had been instructed to draw a picture.

Few viewers in the pilot study could infer the true intent underlying V’s behavior—to keep the knot over the dot. The nature of this discrepancy led us to coin the term *control blindness,* which explains that what viewers were failing to detect was the variable V was controlling because they focused on V’s unintended behavioral consequences. We thought that control blindness might occur only under specifically designed conditions, rather like a visual illusion. But, in fact, the phenomenon was quite robust, leading us to realize that control blindness can reveal important information about the way people make inferences about intention in the same way that visual illusions reveal important information about the way people perceive the environment.

The purpose of this study was to determine (a) the prevalence of this control blindness phenomenon, (b) the types of incorrect inferences about intentions that people make, (c) whether providing the correct answer about a person’s intention can reduce control blindness, and (d) whether merely directing attention toward the controlled aspect of the environment (the knot) reduces the effect. Beyond exploring these aspects, we will also show how PCT, instantiated as a computer model, can be used to discriminate true from apparent intentions, thereby providing a basis for measuring the accuracy of judgments of intention.

## Method

### Participants

#### Study 1

The first sample was gathered during two open days at the university. Participants were recruited for the study, which was billed as an “opportunity of find out about psychology research” as well as a test to determine whether “you can work out what someone is doing by watching what they are doing.” A total of 102 volunteers (57 females, 45 males, *M* = 30 years of age, range: 16–90 years of age) took part. Of these participants, 36 were employed, two were retired, and 64 were students.

#### Study 2

The second sample was gathered online through a Facebook survey to access a wider range of participants. A total of 318 responses were received, but only 236 respondents completed the survey in its entirety (158 females, 78 males, *M* = 40 years of age, range: 16–90 years of age). Of these participants, 20 were in unpaid work, 38 were academic professionals, 17 were technological professionals, 13 were public service professionals, 66 were private sector professionals, 34 were mental health professionals, 17 were non-postgraduate students, and 21 were postgraduate students.

#### Study 3

Participants were recruited through an open day in the same way as Study 1. A total of 81 participants took part, 79 of whom had not seen the demonstration before (37 females, 42 males, *M* = 32 years of age, range: 16–59 years of age). Of these participants, 37 were students, 39 were employed, and three did not provide occupation information.

### Materials

#### Video display

The video depicts the rubber band demonstration as described earlier (available at www.youtube.com/watch?v=Zot0HqETp3U).

### Procedure

#### Study 1

Participants read the information sheet and consented to take part in the study. They were seated in chairs at a comfortable distance from a computer monitor, which displayed the first frame of the video. They then completed demographic information and received a description of the first video: “You will now be shown a video of two people moving pens on a whiteboard. Between the pens is a rubber band. The person on the right is the demonstrator and he has given an instruction to the person on the left. Your task is to guess what the person on the left has been instructed to do. Please view the video now.” Following the reading, participants viewed the 32-second-long video clip for the first time. The video depicts, as mentioned above, two pens connected by rubber bands moving on a whiteboard. If a participant requested another viewing of the clip, this was permitted.

After viewing the video clip, participants completed two open questions: “What has the person on the left been instructed to do?” “Please tell us what you noticed that made you give the previous answer? If you have been told the answer to this test on a previous occasion, please state this here.”

Next, the participants received one interpretation for what the person on the left had been instructed to do, which was on a separate page from the open-ended questions: “One answer to this test is that the person on the left is trying to keep the knot in the middle of the rubber band immediately above a dot that is located in the middle of the whiteboard.” Note that this interpretation was indeed what the person on the left in the video had been instructed to do.

Participants viewed the video again, and were asked: “Having looked at the video a second time, how likely is this new explanation to be correct?” rating from 1 (*not at all likely to be correct*) to 5 (*extremely likely to be correct*). The second question asked how familiar the participant was with PCT, from 0 (*not at all familiar*) to 5 (*extremely familiar*). The study took between 4 and 7 minutes to complete.

#### Study 2

The procedure was similar to that of Study 1 with some minor alterations. After reporting their demographics, the online sample was asked whether they had seen the rubber band demonstration before (Yes/No). Furthermore, because of the small number of participants in Study 1 who said that they did not see the knot of the rubber band, the description of the video was altered slightly: “Between the pens is a rubber band *with a knot in the middle . . .* .” Italics were not reproduced in the study.

#### Study 3

This final study sought to address lingering concerns from the first two studies. It explored whether attending toward the perceptual variable being controlled (the knot position) raised the proportion of correct inferences. Participants were presented with identical conditions and instructions to Study 1 with the exception of the instruction concerning where to focus their attention. A spreadsheet randomizing function was used to randomly allocate each individual to either the “knot” condition (*n* = 40) or the “pen” condition (*n* = 39). The knot group was told, “Your task is to watch the movement of the knot of the rubber band in the video as closely as possible because you will be asked about this at the end of the video.” In contrast, the pen group was told, “Your task is to watch the movement of the pen of the person on the left in the video as closely as possible because you will be asked about this at the end of the video.” A manipulation check involved asking participants to rate how much of the time they had spent looking at the movement of the knot of the rubber band as well as how much time they had spent looking at the movement of the pen (0 = *none of the time* to 10 = *all of the time*).

### Analysis

Open-ended answers for the three samples were coded by two independent researchers (Study 1: 89% accuracy, Kappa = .85; Study 2: 97% accuracy, Kappa = .91; Study 3: 100% accuracy, Kappa = 1.00; see Table 1). For mismatches, the coded answer was agreed upon by the researchers through discussion. The coding methods were the same in Studies 1 and 2, using mutually agreed categorizations, while the responses in Study 3 were coded only for correct versus incorrect,

**Table 1:** The categories of inference regarding the behavior of the volunteer in the video of the rubber band demonstration are shown with the defining criteria and illustrative examples of each category

Category	Defining criteria	Examples
Draw something	To draw or write something; or references an object, animal, or image (e.g., a portrait, a horse, a circle). The other person is not referenced.	“draw a kangaroo boxing with another kangaroo” “draw a map of Crete” “draw a horse” “write their name”
Do the opposite	To do the opposite of the person on the right; to mirror or do the reverse of their actions.	“mimic the person on the right (hand movements) in a mirror reflection around a pivot” “do the opposite of the person on the right”
Copy	To copy or mimic the person on the right; to anticipate or follow the person on the right; to draw or write the same as the person on the right.	“asked to copy the other person” “to draw the same as the person on the right”
Interference	Interference with anything person on the right is trying to do.	“stop the person on the right from drawing something”
Keeping constant	To keep some variable constant (but not to keep the knot over the dot).	“has to react to the right hand to keep the rubber band under strain”
Go with the flow	Let pen or rubber band glide or guide.	“relax and let rubber band guide” “just let the pen glide”
Lead	Lead the movement or make the person on the right follow.	“lead!”
Correct answer	To keep the knot of the rubber band over the dot (or in the center or middle of the page).	“to keep the joining of the rubber bands at the dot”
Multiple	Provide multiple incorrect answers that do not fit under the same category.	“to draw some sort of animal and other person mimicked him” “follow the direction (in and out) of the person on the right, but do the inverse in the up or down direction”
Multiple including correct	As above, with the addition of another inference that is correct.	“draw something and keep the knot in the center as much as he can”

Statistical analyses considered aspects associated with correct versus incorrect answers. In the first and second samples, predictor variables (previously seen [binary]; gender [binary]; age [continuous]; profession [dummy-coded categorical]) were inputted into a binary logistic regression.

## Results

### Study 1

**Fig. 3:**
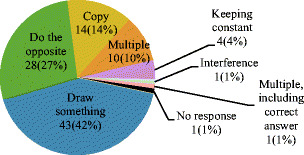
Number and proportion of inferences from Study 1 by category

In our first test of control blindness, we asked 104 participants to view the video of the rubber band demo and describe what V was instructed to do. That is, they were asked to infer the intention underlying V’s behavior in the video. Figure 3 shows the number and proportion of answers for each inference category in Table 1. No participant correctly inferred V’s goal of keeping the knot over the dot. The majority (94%, *n* = 96) of the participants’ inferences indicated “control blindness,” with unintentional actions being identified as V’s intention. Yet, when later presented with the correct answer, 75% (*n* = 87) rated V’s true intention as highly believable (represented by a believability rating of 4 or 5 out of 5). Familiarity with PCT, the theory that would allow a viewer to correctly infer V’s intention, was very low (*M* = 1.23, *SD* = 0.61).

### Study 2

**Fig. 4:**
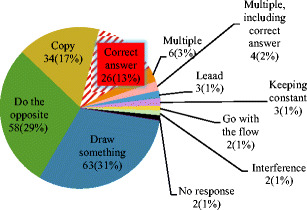
Number and proportion of inferences from Study 2 by category for the participants who had not seen the rubber band demonstration before

Figure 3 shows the number and proportion of answers from each category in an online study with respondents who reported not having seen the demonstration before (*n* = 203, out of a total *N* = 236). A minority of these (13%, *n* = 26) provided the correct answer, and the majority (84%, *n* = 170) provided answers fully consistent with having experienced control blindness (see Fig. 4). However, as in Study 1, the majority of respondents (68%, *n* = 139) reported high believability scores after viewing the correct inference. When the entire sample’s data (*N* = 236) was analyzed, a regression model, χ^2^(10) = 63.11, Cox-Snell *R* ^2^ = .24, *p* < .001, indicated that a correct answer was associated with having seen the demonstration before, B = 2.99, *SE* = .57, Wald = 27.48, *df* = 1, *p* < .001, Exp (B) = 19.81, a younger age, B = -0.04, *SE* = .02, Wald = 4.22, *df* = 1, *p* < .05, Exp (B) = 0.97, and being male, B = 1.15, *SE* = .39, Wald = 8.83, *df* = 1, *p* < .01, Exp (B) = 3.16, but not with profession, Wald = 2.72, *df* = 7, *ns*.

### Study 3

As expected, participants in the knot condition reported spending longer looking at the knot than those in the pen condition (knot: *M* = 4.78, *SD* = 3.23; pen: *M* = 1.03, *SD* = 1.46), *t*(77) = 6.62, *p* < .001. Similarly, participants in the pen condition reported spending more time looking at the pen than those in the knot condition (pen: *M* = 7.59, *SD* = 1.41; knot: *M* = 4.30, *SD* = 2.99), *t*(77) = 6.23, *p* < .001. Therefore, the attention manipulation was considered to have been successful. In the knot condition, five participants correctly identified V’s intention to keep the knot over the dot, and 35 participants were incorrect. In the pen condition, four participants were correct, and 35 participants were incorrect. A chi-squared test showed there to be no effect of this attentional manipulation on correct inferences of intention, χ^2^(1) = .00, *ns*.

### Computer model

We developed a computer model of V’s behavior in the rubber band task to show that what V was instructed to do—V’s goal—was *only* to control the distance between knot and dot. That is, the model will show that V’s only intention was to keep the knot over the dot; the pattern traced out by V’s pen movement was an unintended side effect of carrying out this intention, just as in the real task, and thereby providing a form of ecological validity.

The model consisted of two control systems, one controlling a perceptual variable, p_x_, that corresponds to the distance from knot to dot in the x dimension and the other controlling a perceptual variable, p_y_, that corresponds to the distance from knot to dot in the *y* dimension. These perceptions were defined as:

1 $$ {\mathrm{p}}_{\mathrm{x} = }{\mathrm{k}}_{\mathrm{x}} - {\mathrm{d}}_{\mathrm{x}}\ \mathrm{and}\ {\mathrm{p}}_{\mathrm{y}} = {\mathrm{k}}_{\mathrm{y}} - {\mathrm{d}}_{\mathrm{y}} $$

where k_x_ and k_y_ are the time varying x and y position of the knot and d_x_ and d_y_ are the constant x and y position of the dot, respectively. Since d_x_ and d_y_ are constants, they can be set to zero so that the position of the dot defines the origin of the x, y coordinate space in which the rubber band demo takes place. So the perceptual variables controlled by V can be defined as p_x =_ k_x_ and p_y_ = k_y_.

The next step is to describe the causes of variation in the position of the knot. Since the knot is attached to rubber bands pulled by both D and V, the position of the knot in the x and y dimensions can be described by the following equations:

2a $$ {\mathrm{k}}_{\mathrm{x}} = {\mathrm{ov}}_{\mathrm{x}} + {\mathrm{od}}_{\mathrm{x}} $$

2b $$ {\mathrm{k}}_{\mathrm{y}} = {\mathrm{ov}}_{\mathrm{y}} + {\mathrm{od}}_{\mathrm{y}} $$

where ov_x_ and ov_y_ are V’s outputs—the time varying x and y positions of V’s end of the rubber band (and, thus, V’s pen)—and od_x_ and od_y_ are D’s outputs, which are the time varying x and y positions of D’s end of the rubber band (and D’s pen). D’s outputs, od_x_ and od_y_, are *disturbances* to the position of the knot from V’s perspective in the sense that they are influences on the position of the knot that are independent of those produced by V.

Finally, the model must specify how the control systems convert perceptions of knot position, p_x_ and p_y_, into the outputs, ov_x_ and ov_y_, that keep the knot over the dot. This was done using the following equations, written as computer pseudocode:

3a $$ {\mathrm{ov}}_{\mathrm{x}} = {\mathrm{ov}}_{\mathrm{x}} + {\mathrm{slow}}_{\mathrm{x}}*\ \left({\mathrm{gain}}_{\mathrm{x}}*\ \left({\mathrm{ref}}_{\mathrm{x}} - {\mathrm{p}}_{\mathrm{x}}\right) - {\mathrm{ov}}_{\mathrm{x}}\right) $$

3b $$ {\mathrm{ov}}_{\mathrm{y}} = {\mathrm{ov}}_{\mathrm{y}} + {\mathrm{slow}}_{\mathrm{y}}*\ \left({\mathrm{gain}}_{\mathrm{y}}*\ \left({\mathrm{ref}}_{\mathrm{y}} - {\mathrm{p}}_{\mathrm{y}}\right)\ \hbox{--}\ {\mathrm{ov}}_{\mathrm{y}}\right) $$

where slow_x_ and slow_y_, gain_x_ and gain_y_, and ref_x_ and ref_y_ are parameters of the model; ref_x_ and ref_y_ are each system’s reference specification for p_x_ and p_y_, the perceived location of the knot; slow_x_ and slow_y_ are slowing factors that determine how quickly ov_x_ and ov_y_, the outputs, change over time (each iteration of the equations). Finally, gain_x_ and gain_y_ are gain factors that determine how much output is produced at each instant per unit error (i.e., the difference between the reference and perceived position of the knot, ref_x_ - p_x_ and ref_y_ - p_y_).

To generate the evidence in support of V’s true goal, the model first needed to be implemented using spreadsheet calculations. The input to the model was the time varying x, y positions of D’s end of the rubber band, od_x_ and od_y_. These positions were derived from the frames of the video of the rubber band demonstration (e.g., Fig. 1). Other variables measured from the video frames were the x, y positions of the knot as well as V’s end of the rubber band.

**Fig. 5:**
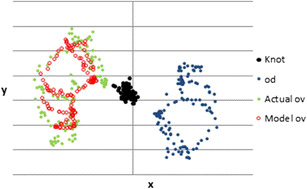
Comparison of V’s actual pen movement behavior (*Actual ov*) to the Model V’s (*Model ov*) pen movement behavior in the rubber band demonstration video. Also shows the behavior of the *knot* and of D’s (*od*) pen movement behavior, the latter being a disturbance to the perception of the distance from knot to dot that V is controlling

The results of the modeling are shown in Fig. 5. The result of particular interest was the fit of the model variations in pen position (Model ov) to V’s actual variations in pen position (Actual ov). The model was fit using a Monte Carlo method of varying the six model parameters (slow_x_, slow_y_, gain_x_, gain_y_, ref_x_, ref_y_) to minimize the squared deviation between model and data. The fit that was achieved was extremely good. The correlation between Model and Actual ov values was *r* = .98. So the model pen movements (Model ov) accounted for 97% (*R*^2^ = .97) of the variance in V’s actual pen movements (Actual ov). The RMS deviation of Model ov from Actual ov values was 5.1, measured in pixels. The total range of Actual ov values was 154.5 pixels, so the model was accurate to within 3% of the actual ov values.

The fit of the model was particularly sensitive to the values selected for reference signal parameters, ref_x_ and ref_y_. The initial assumption was that both of these parameters should be set to zero since V was instructed to keep the knot over the dot, which was defined as the 0, 0 position in the video. With this setting, the *R*^2^ measure of fit was still .97, but the RMS deviation of model from actual ov’s, measured as percentage to total possible deviation, was 7%—more than twice what could be achieved with ref_x_ = -32 and ref_y_ = 10. This led to the realization that V was controlling a parallax view of the distance from knot to dot, so that the perception of knot position would be seen as over the dot when the actual knot position was offset from the dot.

Note that the pattern traced out by the model pen movements (Model ov) in Fig. 4 is almost exactly the same as that traced out by V (Actual ov). This suggests that the pattern traced out by V is not V’s intention, although the majority of viewers of the video concluded that some aspect of this pattern was. The model did not have the goal of producing any particular pattern of movements; it had no reference for the pattern that resulted from its pen movements. The model’s only goal was to keep perceptions of the distance of knot to dot close to the references for these perceptions. To do this, the model had to produce pen movements (outputs, ov_x_ and ov_y_) that were equal and opposite to the disturbances to these perceptions created by D’s pen movements (od_x_ and od_y_). Thus, like the pattern of V’s actual pen movements, the pattern of model V’s pen movements was the mirror image of the pattern of D’s pen movements. This illustrates that the mirroring behavior was unintended since the model could not “see”—and therefore could not intentionally imitate—the pen movements that were being mirrored. The mirroring was a side effect of achieving the goal of keeping a perception of the knot over the dot; it was not one of V’s goals.

In addition to providing strong support that V controls the knot’s configuration, the modeling exercise shows that it is a *perception* of the distance from knot to dot and not the actual distance that is being controlled. This was made apparent when we found that the values for the parameters for the reference signals in the model, ref_x_ and ref_y_, that gave the best fit of model to actual pen movements were -32 and 10, respectively, rather than zero and zero. This implied that V’s intention was to keep the knot somewhat to the left and above the dot, which was not what V was instructed to do. However, given the angles of V’s vision, V perceived the knot and dot to be aligned when the knot was displaced to the left and slightly below of the central dot. To keep the perception of the knot over the dot, V would have to keep the actual position of the knot (the x, y position derived from the video frames) in a position that “compensated” for this parallax.

## Discussion

Our results suggest that people tend to attribute intention to aspects of behavior that appear to be intentional, but which are not. The pattern drawn by the volunteer was an unintended side effect of the true intention, which was to control the position of a knot in rubber bands relative to a target dot beneath the rubber bands. Seeing the unintentionally produced pattern of pen movement as intentional (controlled) is an example of what has been called “the illusion of control” (Langer, 1975). This study shows that the side effects of intentional behavior that create the illusion of control can be so compelling that they blind people to the true intention, leading to *control blindness*.^1^ We have used this term because it appears that viewers are simply unable to see the process of control as it is occurring in this demonstration and instead see the actions that achieve it. We tested this directly by manipulating attention toward the controlled knot, and the effect is seen to remain. Second, the term is somewhat analogous to the widely cited phenomenon of *change blindness* (Simons & Chabris, 1999). People are typically aware of change in their environment, and yet certain experimental conditions that manipulate awareness through a concurrent task lead individuals to miss changes (e.g., a gorilla walking through a basketball match; Simons & Chabris, 1999) that would otherwise be easily noticed. Similarly, people may recognize control occurring in everyday situations, but the experimental conditions of the rubber band demonstration led them to miss control as it is occurring. However, we found that control blindness was unlikely to be the result of inattention as in the change blindness effect because successfully guiding attention to the knot did not diminish the effect.

This study differs in several ways from previous of studies of the accuracy of inferences of intention (Baker, Saxe, & Tenenbaum, 2009; Pantelis et al., 2014). First, other studies used simulated agents that did not necessarily produce ecologically valid examples of intentional behavior. We used a real-life example of intentional behavior—control—and tested its ecological validity (“ground truth”) using a computer model. Second, in other studies of the accuracy of judgments of intentionality, the range of response to identify possible intentions was limited. Thus, these studies did not assess the naturalistic tendency for people, unaided by suggestions from the experimenter, to make inferences regarding intention. While we see the validity of such research in answering the question of whether different intentions are perceived accurately, these studies did not have the capacity to examine whether unintentional actions can be perceived as intentional; the only possibility was to see the actions as exhibiting a certain intention. In this study, participants were not limited in the intentions that could be identified. Thus, it was possible to correctly identify V’s true intention or falsely identify unintended results of actions as intended (which was usually the case).

A full analysis of intentional behavior based on PCT would involve a hierarchy of goals (Powers, 1973). For example, the goal of “keeping the knot over the dot” would be subordinate to the goal of “following the instructions” and superordinate to the goal of “pulling on the rubber band.” But the participants in this study were not asked to identify any intentions other than the one V was instructed to carry out throughout the video: to keep the knot over the dot. Furthermore, the model of V’s behavior shows that the behaviors that participants most often identified as V’s goal—“Copying D,” “Doing the opposite of D,” and “Drawing something”—were actually unintentional side effects of V carrying out the intention of keeping the knot over the dot.

The experimental studies showed that proneness to control blindness was widespread. Control blindness related to age and gender differences, and it also seemed to be largely a consequence of being unable to generate the correct inference. These findings are consistent with those of other effects and illusions, which can be ameliorated by experimental manipulations (Richards, Hannon, & Derakshan, 2010). Yet a sizeable minority of our samples did not believe the correct answer, suggesting that the effect can be p ersistent. Furthermore, our participants’ diverse inferences indicate that they interpreted the unusual pen movements made by the volunteer, to the expense of noticing the control of the knot over the dot. This may explain why earlier studies, in which there is a mapping between more obvious motion cues and (assumed) intentions, appear to find greater accuracy regarding inferences of intention (Barrett et al., 2005; Schachner & Carey, 2013). Yet, as stated earlier, because the effect remained when attention was directed toward the knot of the rubber band, it appears to not be a mere artifact of the focus of attention.

It could be argued that these results are unique to the (simple) rubber band demonstration. Replication in other settings might explore such a concern, namely through testing with a computer simulation (McPhail, Powers, & Tucker, 1992) or a more complex physical apparatus (Shaffer, Marken, Dolgov, & Maynor, 2014). Beyond these concerns, our results are consistent with earlier studies that found low accuracy in judgments of a person’s actual intention, where the person’s intention was conceived as it is in this study—as an aspect of the environment that the person was actually controlling (Jordan & Hershberger, 1990; Marken, 2013b). Neither of these studies used the rubber band demonstration, but they did involve tracking movement. Importantly, in both of these studies, the participants were given a forced choice rather than an open question as in the current study, and the results were the same—the majority made incorrect inferences of intention. This indicates that our findings were unlikely to have arisen because of the open question format of the study. The advantage of such as method was that it did not give participants clues as to the likely intentions, thereby being closer to a naturalistic inference regarding observed behavior. Our findings differ notably, however, from most previous studies of the accuracy of judgments of intention (such as Barrett et al., 2005; Call & Tomasello, 2008) because they provide an objective basis for discriminating intentional from unintentional behavior. The objective basis is provided by PCT, which represents intentional behavior as controlled results of actions (such as the distance from knot to dot in the rubber band demo) and unintentional behavior as uncontrolled side effects of control actions (such as the drawing of the “boxing kangaroos”; Marken, 2013b).

We believe this research has important implications for psychological research (Marken & Mansell, 2013). It suggests that the behaviors that are studied in psychological research may be no more than a side effect of participants’ true intentions. The results of this research suggest that behavior should be defined in terms of its goal; that is, behavior should be defined in terms of the perceptual variables the participants are controlling. Indeed, according to PCT, identification of intentions, which are typically not obvious at all (as in the rubber band demonstration), should be the main goal of research aimed at understanding behavior. This entails that behavior itself becomes a defining feature of cognition and cannot be considered separately (Gomez-Marin & Mainen, 2016). These goals (theory of mind) can be inferred objectively using methods derived from PCT (Marken, 2013a). Our aim was not to try to explain *how* observers make accurate inferences of intention, although this may form the basis of future research. It certainly appears that people are prone to impute purpose to others, and some accounts suggest that the viewer may use a mental simulation of themselves engaged in the action in order to make the inference of intention (e.g. Pezzulo & Castelfranchi, 2009). Interestingly, the PCT architecture specifies the operation of an imagination mode that engages in mental simulation (Powers et al., 1960). In future research, this mode could be implemented in order to model not only the actor but also the observer of the purposeful action.

The results of this research further suggest that control blindness may create an obstacle for advances in research, policy, and public interventions that propose people’s behavior can be changed through manipulating environmental “triggers” (Michie, van Stralen, & West, 2011). Environmental events, such as D’s pen movements in the rubber band demonstration, can appear to trigger (cause) behavior, such as V’s pen movements, when the true intention of V’s behavior goes unnoticed. PCT shows that the apparent causal connection between environmental triggers (stimuli) and behavior (responses) is an illusion (Powers, 1978). The PCT model of behavior suggests that policies and interventions should be aimed at helping people achieve their own goals rather than providing them with the best “triggers” for action. We therefore plan further studies of the effects of control blindness specifically within these contexts.

## Footnotes

1 Because V’s unintentional pen movements were consistently seen as intentional may be explained by the empirical Bayesian inference process posited by active inference theory (Kilner et al., 2007; Friston, Mattout, & Kilner, 2011).
